# Clinical and Epidemiological Characteristics of Scrub Typhus and Murine Typhus among Hospitalized Patients with Acute Undifferentiated Fever in Northern Vietnam

**DOI:** 10.4269/ajtmh.14-0806

**Published:** 2015-05-06

**Authors:** Sugihiro Hamaguchi, Ngo Chi Cuong, Doan Thu Tra, Yen Hai Doan, Kenta Shimizu, Nguyen Quang Tuan, Lay-Myint Yoshida, Le Quynh Mai, Dang Duc-Anh, Shuji Ando, Jiro Arikawa, Christopher M. Parry, Koya Ariyoshi, Pham Thanh Thuy

**Affiliations:** Department of Clinical Tropical Medicine, Institute of Tropical Medicine, Graduate School of Biomedical Sciences, Nagasaki University, Nagasaki, Japan; Infectious Disease Department, Bach Mai Hospital, Hanoi, Vietnam; Departments of Virology I, National Institute of Infectious Diseases, Tokyo, Japan; Laboratory of Infectious Diseases, Department of Microbiology, Hokkaido University Graduate School of Medicine, Hokkaido, Japan; National Institute of Hygiene and Epidemiology, Hanoi, Vietnam; Department of Clinical Research, London School of Hygiene and Tropical Medicine, London, United Kingdom

## Abstract

A descriptive study on rickettsiosis was conducted at the largest referral hospital in Hanoi, Vietnam, to identify epidemiological and clinical characteristics of specific rickettsiosis. Between March 2001 and February 2003, we enrolled 579 patients with acute undifferentiated fever (AUF), excluding patients with malaria, dengue fever, and typhoid fever, and serologically tested for *Orientia tsutsugamushi* and *Rickettsia typhi*. Of the patients, 237 (40.9%) and 193 (33.3%) had scrub and murine typhus, respectively, and 149 (25.7%) had neither of them (non–scrub and murine typhus [non-ST/MT]). The proportion of murine typhus was highest among patients living in Hanoi whereas that of scrub typhus was highest in national or regional border areas. The presence of an eschar, dyspnea, hypotension, and lymphadenopathy was significantly associated with a diagnosis of scrub typhus (OR = 46.56, 10.90, 9.01, and 7.92, respectively). Patients with murine typhus were less likely to have these findings but more likely to have myalgia, rash, and relative bradycardia (OR = 1.60, 1.56, and 1.45, respectively). Scrub typhus and murine typhus were shown to be common causes of AUF in northern Vietnam although the occurrence of spotted fever group rickettsiae was not determined. Clinical and epidemiological information may help local clinicians make clinical diagnosis of specific rickettsioses in a resource-limited setting.

## Introduction

Rickettsial infection is one of the common causes of acute undifferentiated fever (AUF) in Southeast Asia after malaria, dengue fever, and typhoid fever have been excluded.^1^,^2^ There are three major rickettsiae causing disease: *Orientia tsutsugamushi*, the pathogen of scrub typhus, *Rickettsia typhi*, the pathogen of murine typhus, and the spotted fever group rickettsiae (SFGR). They are transmitted by arthropods, the larval stage of trombiculid mites, the oriental rat flea (*Xenopsylla cheopis*), and mainly the larval to adult stage of ticks, respectively, and the infection cycles are maintained by the mite itself for *O. tsutsugamushi*, rats and fleas for *R. typhi*, and animal hosts and ticks for the SFGR.^3^ Therefore, disease distribution is largely determined by the distribution of these vectors and reservoirs.^3^

An accumulating number of studies have reported that scrub typhus is present in most countries in the Southeast Asia, including Thailand, Cambodia, Laos, Bangladesh, Indonesia, and Vietnam.^4^–^11^ Detailed information relating to the epidemiology and clinical characteristics of each rickettsial infection remains limited. Most published studies have been confined to Malaysia, Thailand, and Laos. Little is known about the clinical epidemiology of murine typhus and none of the rickettsiae has been fully investigated in northern Vietnam.^12^

The clinical presentation of rickettsial diseases ranges from a mild, non-specific febrile syndrome to a life-threatening fatal condition. They may mimic tropical febrile illnesses such as malaria, dengue fever, typhoid fever, and leptospirosis.^13^ In particular, murine typhus is likely to be underdiagnosed or to be confused with a viral illness because patients usually do not recognize transmission from fleas and the majority of cases resolve spontaneously.^14^ There is no reliable point-of-care laboratory test for rickettsial disease. Even in a referral hospital in *Rickettsia*-endemic countries, the diagnosis of rickettsioses is usually based on the clinical findings.^15^,^16^ Most clinical studies on rickettsioses to date have focused on the introduction and evaluation of laboratory diagnostic techniques.^17^ Few studies have characterized the clinical picture of different rickettsiosis and none attempted to calculate odds ratio (OR) of clinical findings for the purpose of differentiating specific rickettsial infections.

More detailed clinical information could lead to improvements in the diagnosis of rickettsioses in resource-limited countries and increase clinician's confidence in managing patients with AUF based on a clinical diagnosis. We have conducted a retrospective investigation with specific objectives of determining the clinical epidemiology of scrub typhus and murine typhus in northern Vietnam and identifying clinical features associated with specific rickettsioses. A qualitative description of the scrub typhus patients in this cohort has been recently published.^12^

## Materials and Methods

### Study design, site, entry criteria, and data collection.

A descriptive study was conducted at the Infectious Disease Department of Bach Mai Hospital in Hanoi, Vietnam. This is the largest referral medical center with approximately 1,900 beds, covering residents in all provinces in northern Vietnam. The area is not malaria-endemic as fewer than 10 malaria cases are hospitalized annually in this hospital and they are either referred from outside northern Vietnam or imported from other countries (data not shown).

Between March 2001 and February 2003, serum samples were collected both in acute and convalescent phases from hospitalized patients suspected of rickettsioses. Patients were enrolled when they fulfilled three primary criteria: 1) aged 15 years or older; 2) having had a documented acute fever, 37.5°C or higher by axillary temperature measurement on and around the admission day without an apparent focus of infection after an initial evaluation of medical history, physical examination, and basic laboratory tests (complete blood counts and basic chemistry profiles); and 3) having had at least one of the following five secondary findings: nonspecific rash, multiple lymphadenopathy, eschar, hepatomegaly and/or splenomegaly, and no recovery after β-lactam antibiotic use. Patients diagnosed with malaria, dengue fever, and typhoid fever, after the initial assessment based on blood smear, blood culture, or strong clinical suspicion, were not enrolled.

In the current study, we systematically cleaned and reanalyzed the patient's clinical information that had been collected at the time of admission using a standardized form. The collected data included background information (age, gender, living areas, occupation, etc.); prescription information; symptoms (duration of fever, headache, myalgia, rigor/chill, cough, sputum, dyspnea, etc.); physical signs (body temperature, heart or pulse rate, respiratory rate, blood pressure, rash, lymphadenopathy, eschar, etc.); laboratory results (complete blood counts, liver enzymes, blood urea nitrogen [BUN], creatinine, etc.); antibiotics prescribed; and patient outcomes (full recovery, death, self-discharge, etc.). We defined the rainy season as the period between May and October in northern Vietnam and defined a high-exposure occupation as an occupation that involved frequent contacts with the natural environment: farming, dairy husbandry, and environmental construction engineering. Relative bradycardia was defined as less than 10 per minute increase in heart or pulse rate when the body temperature increased 1°C.^18^ Rash, lymphadenopathy, and edema in our study were defined as non-localizing and nonspecific characteristics.

### Laboratory tests.

Serum samples were first screened in 2005 with a commercially available IgM enzyme-linked immunosorbent assay (ELISA) for *O. tsutsugamushi*, which measures IgM antibodies against the 56 kDa outer membrane protein (PanBio, Alere, Australia). A positive result was defined according to the instruction of the product company. The ELISA negative samples of patients registered in the first year were further tested in 2008 with a commercially available IgG immunofluorescent assay (IFA) kit for *R. typhi* (Focus Diagnostics, Cypress, CA), which measures IgG antibodies against the *R. typhi* antigen. To complete the data set, we tested remaining serum samples of patients registered in the second year with in-house IgG IFA in 2013. In the in-house IFA, antigens of *R. typhi* (strain Wilmington) were used. In both commercial and in-house IgG IFA test, positive result was defined by either a high titer of ≥ 400 in single sample or ≥ 4-fold increase in titer in paired samples.^19^

### Statistical analysis.

Categorical variables were summarized as frequencies and percentages, and the χ^2^ test or Fisher exact test was used for the comparison of clinical characteristics among different groups. Continuous variables were summarized as mean and standard deviation, and Student's unpaired *t* test was used for two-group comparison. A logistic regression analysis was used to produce ORs. All tests were two-tailed and *P* < 0.05 was regarded as statistically significant. STATA version 13 (StataCorp LP, College Station, TX) was used for statistical analysis.

### Ethical consideration.

Verbal informed consent was obtained from all the patients in the original enrollment. In 2011, with strict personal information protection as conditions, institutional review boards and independent ethics committees of both Bach Mai Hospital and Institute of Tropical Medicine, Nagasaki University, approved this retrospective study and the retrospective use of the patient information.

## Results

### Investigation flow.

**Figure 1.**
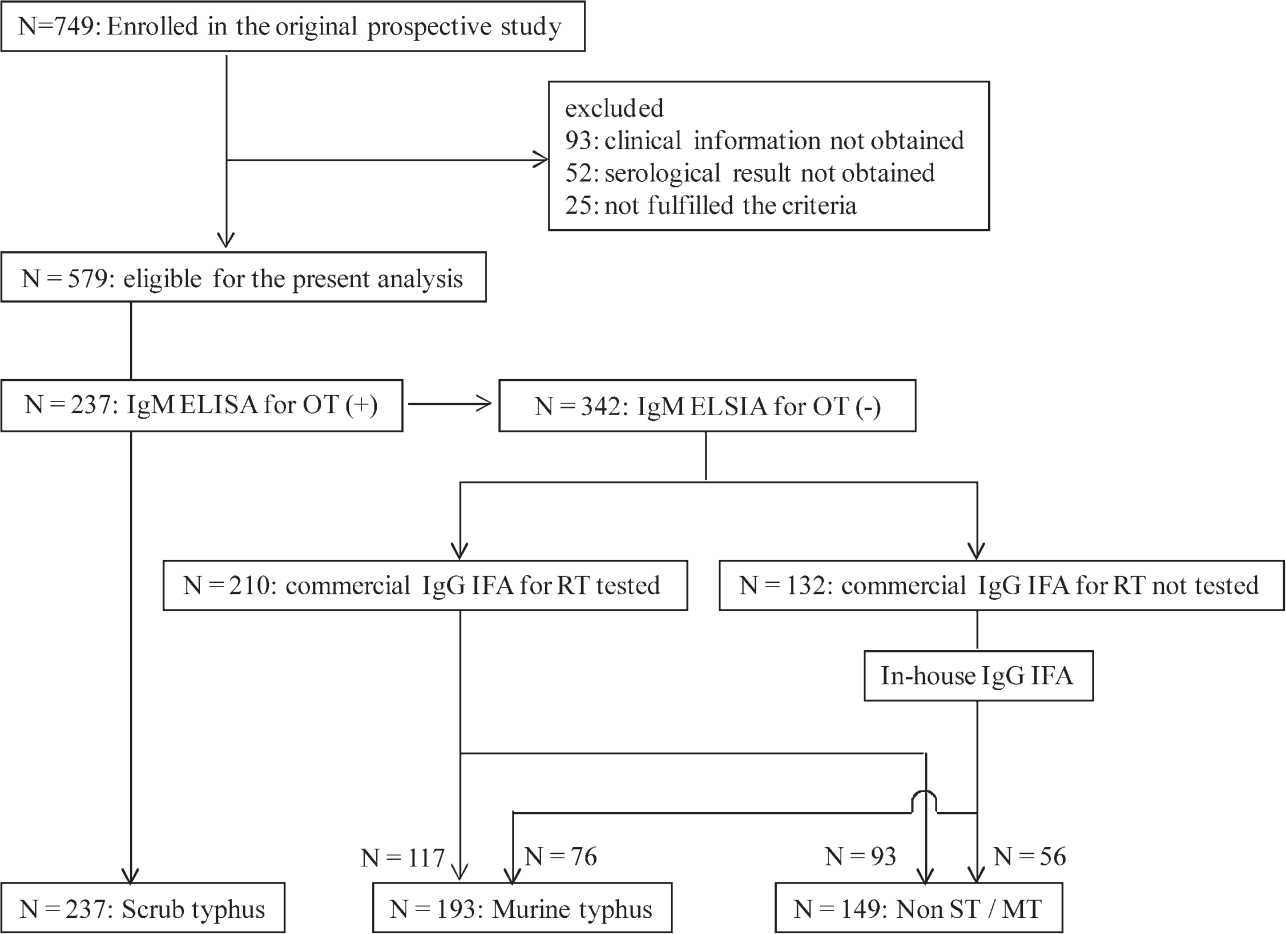
Investigation flow of patients in the study. OT = *Orientia tsutsugamushi*; RT = *Rickettsia typhi*; non-ST/MT = non–scrub and murine typhus; ELISA = enzyme-linked immunosorbent assay; IFA = immunofluorescent assay.

There were 749 patients whose serum samples were collected between March 2001 and February 2003. Clinical information and scrub typhus serology results were retrieved from 579 patients but 170 were excluded from the analysis because 93 had no clinical information, 52 had no serology results, and 25 did not fulfill the entry criteria. There were no significant difference in the background characteristics such as sex, living in Hanoi, rainy season, risky occupation, ineffective with β-lactam antibiotics, rash, lymphadenopathy, eschar, and hepatomegaly and/or splenomegaly in 52 patients without serology data, compared with 579 included patients except the mean age of the excluded patients was significantly older (Supplemental Table 1). There were 237 patients positive for *O. tsutsugamushi* in the IgM ELISA. Out of the remaining 342 patients with a negative ELISA, 210 patients were tested with commercially available IgG IFA for *R. typhi* and 117 patients were positive. Among 132 patients who were not tested with commercially available IgG IFA, 76 patients were positive for *R. typhi* tested with in-house IgG IFA and 56 patients were negative. To confirm that it was reasonable to combine the results of the IFA assays, we tested samples from 24 patients by both the in-house IFA assay and the commercial IFA assay and found 100% concordance. Consequently, a total of 579 patients were classified into scrub typhus (*N* = 237, 40.9%), murine typhus (*N* = 193, 33.3%), and neither of them (non-ST/MT; *N* = 149, 25.7%) (Figure 1

).

### Patient demographic and clinical information.

**Figure 2.**
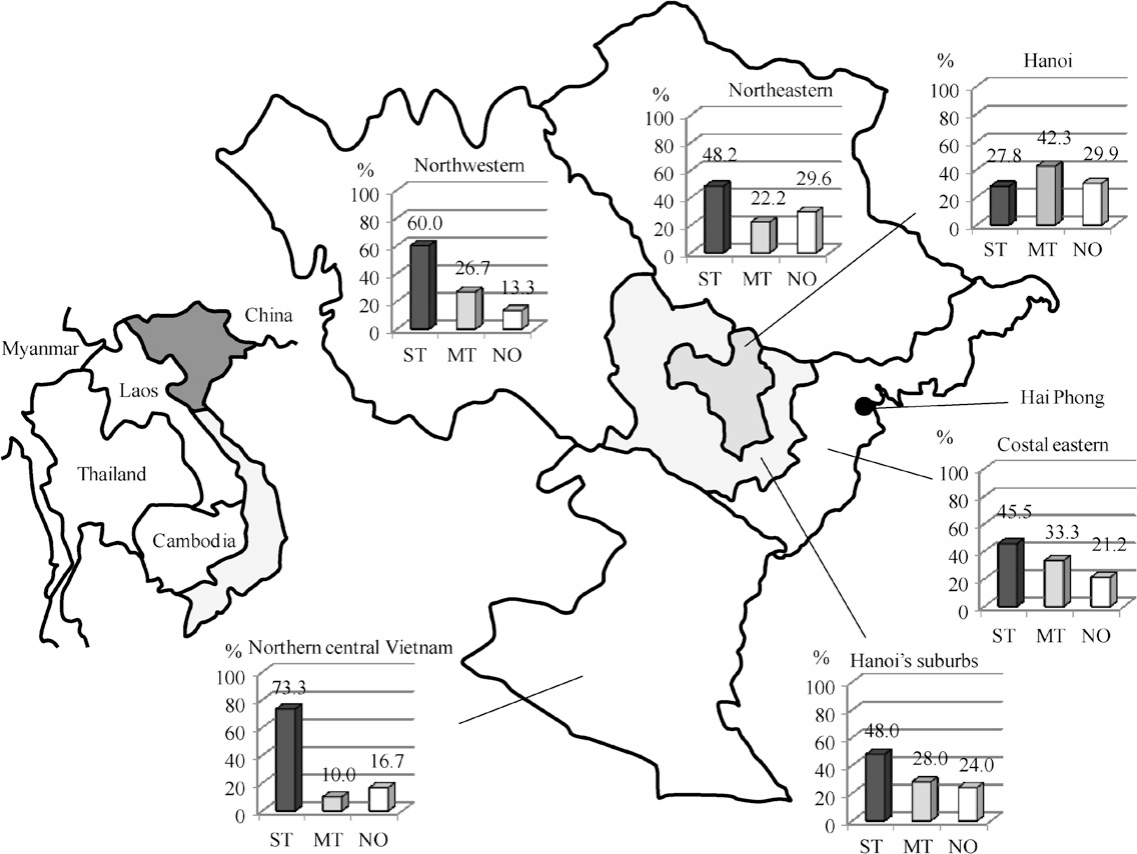
Regional demographics of proportion of patients with scrub typhus, murine typhus, and non-scrub and murine typhus. ST = scrub typhus; MT = murine typhus; NO = non–scrub and murine typhus.

**Figure 3.**
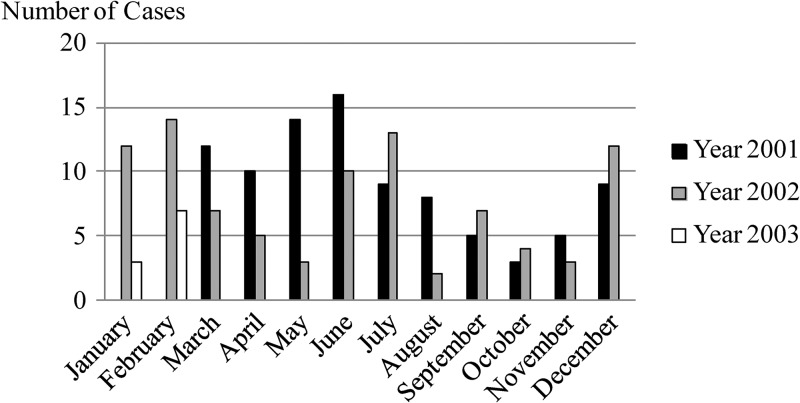
Seasonality of frequency of murine typhus.

The demographic and clinical features of the patients are given in Table 1. The mean (standard deviation) age of the 579 patients was 46.2 (15.7) years, and of them 358 (61.8%) were male. There were no significant differences in the mean ages in different diagnostic groups but patients with murine typhus were more commonly male. There were differences in the geographic distribution of each group. The proportion of murine typhus was highest among patients living in Hanoi (42.3%), followed by costal eastern (33.3%) whereas that of scrub typhus was high in northern central and northwestern areas (73.3% and 60.0%, respectively) (Figure 2

). The number of patients with scrub typhus showed a peak during the rainy season as described in the previous report,^12^ but patients with murine typhus did not have a clear seasonality (Figure 3

).

Among the 244 patients who had received antibiotics before admission, 189 (77.5%) patients had been treated with β-lactam antibiotics without clinical improvement. The majority of patients had fever more than 1 week, and also had systemic constitutional symptoms such as myalgia, chills or rigor rather than organ-specific symptoms. An eschar was reported in 62.9% patients with scrub typhus and some patients in the other groups also had eschar (murine typhus, 2.1%; non-ST/MT, 5.4%). Almost all patients with scrub typhus and murine typhus had elevated liver transaminases, but not in the non-ST/MT patients. The recovery duration after treatment was short in most cases. Only one patient died, who had scrub typhus (Table 1). This patient presented to the hospital 10 days after the symptom onset and had acute respiratory distress syndrome.

### Quantification of clinical findings for scrub typhus, murine typhus, and non–scrub and murine typhus.

We compared the clinical findings between one group and the other two groups, and quantified the strength of diagnostic factors using ORs as effect size (Table 2). Patients with scrub typhus had eschar with the highest OR (46.56, 95% confidence interval (CI): 24.71–87.72), followed by dyspnea (10.90, 95% CI: 4.19–28.38), hypotension (9.01, 95% CI: 2.00–40.65), lymphadenopathy (7.92, 95% CI: 5.41–11.59), altered mental status (5.14, 95% CI: 2.17–12.19), and edema (4.50, 95% CI: 2.77–7.31). Patients with murine typhus were significantly less likely to have these symptoms and signs, but more likely to have myalgia, rash, and relative bradycardia (OR = 1.60, 1.56, and 1.45, respectively). Patients with non-ST/MT were less likely to have elevated transaminases, prolonged fever, elevated BUN, and leukocytosis with an OR of 0.24, 0.45, 0.47, and 0.51, respectively.

## Discussion

This is the first study to demonstrate the common presence of murine typhus in addition to scrub typhus among AUF patients in northern Vietnam. One-third AUF patients, after malaria, dengue fever, and typhoid fever had been excluded on the basis of a malaria smear, blood culture, or clinical findings, had murine typhus. We further clarified the difference in clinical and demographic findings between the two major rickettsioses by calculating OR. We believe that our findings will help local physicians to clinically distinguish among patients with scrub typhus, murine typhus, and non-ST/MT.

Several other published papers have reported a high prevalence of eschar among scrub typhus patients but most studies did not analyze the strength of its diagnostic value. The current study quantitatively demonstrated that the presence of eschar was the most important diagnostic clue for scrub typhus with an OR of 46.56. Our results should be interpreted carefully because the prevalence of eschar varies among different studies, and some patients with SFGR also present with eschar.^20^–^22^ In our study, four patients classified as murine typhus and eight patients classified as non-ST/MT had an eschar. They may have had SFGR, but these agents were not systematically screened for in this study. The positive serology for *R. typhi* may also be due to a cross-reaction with SFGR antibodies. Furthermore, about one-third of our patients with serologically confirmed scrub typhus did not have identifiable eschar. A combination of other factors is, therefore, necessary for the clinical diagnosis of scrub typhus with no eschar. Respiratory symptoms, hypotension, and altered mental status have been reported to be associated with the severe form of scrub typhus.^23^,^24^ Lymphadenopathy is described as well-known clinical signs of scrub typhus in other studies.^10^,^25^ Edema is thought to be due to hypoalbuminemia, which is reported as an outcome marker for complications.^26^,^27^ Although a rash is known to be one of the typical findings of scrub typhus, patients with rash were less likely to have scrub typhus in our study. Rash reportedly appears early in the course of the disease and persists for a short duration.^10^ The majority of our patients with scrub typhus came from outside Hanoi and had fever for more than 7 days. We hypothesize that the rash in some patients could have disappeared by the time of attendance to the hospital.

Murine typhus is a more challenging disease to diagnose. This is because the disease is mostly self-limiting, and no specific findings have been reported.^14^ In this study the presence of myalgia, rash, and relative bradycardia and the absence of significant findings of scrub typhus may support clinical diagnosis of murine typhus. A confirmed diagnosis of scrub or murine typhus was associated with elevated liver enzymes consistent with other reports.^28^,^29^ Histopathological changes in the liver of patients with scrub and murine typhus have been identified in case reports.^30^,^31^

In addition to the clinical findings, our results showed that epidemiological information contributes to the diagnosis of rickettsioses. Patients with scrub typhus were more likely to live in the areas of country or regional borders, whereas patients with murine typhus were more likely to live in Hanoi or coastal provinces including Hai Phong. One study has reported that several rat-borne pathogens were identified from rats in Hai Phong, one of the largest ports in northern Vietnam, although *R. typhi* was not specifically tested.^32^ Seaports are known to be a major source of rodent-borne illness.^33^ The rainy season is coincident with rice field work in northern Vietnam, and rainfall is reported to be strongly associated with larval mite population in the lifecycle.^34^ We think that this host–vector situation might explain the highest incidence of scrub typhus in this season. Rapid clinical improvement after the initiation of effective antibiotics is also reported to backup clinical diagnosis of rickettsioses.^35^,^36^ This was not clear in our study because we were unable to obtain precise information concerning recovery times after initiation of effective antibiotics such as doxycycline although we do know that more than three quarters of the patients recovered within 3 days after hospitalization. The combination of our results and the existing knowledge such as rapid response to effective antibiotics will aid in the clinical diagnosis of rickettsioses.

The prevalence of confirmed *Rickettsia* diagnoses in the current study was extremely high, at 74.3%, compared with previous studies, reporting the prevalence of 5.0–17.6% even after the exclusion of malaria.^37^,^38^ This high prevalence may be not only because rickettsioses is a major cause of AUF in northern Vietnam but also because our inclusion criteria, which were primarily aimed to recruit patients with rickettsioses, were appropriate for this purpose. We sought to exclude not only patients with malaria but also those with dengue and typhoid fever from the analysis albeit by clinical suspicion. Furthermore before the current study was conducted, the awareness of rickettsioses among clinicians in northern Vietnam was low and doxycycline was not always available in district hospitals. Consequently, patients who did not respond to β-lactam antibiotics were more likely selectively referred to this hospital. Since the discovery of this high prevalence rate, the information was circulated among local clinicians; thus, the situation may have changed by now.

Our study has some limitations. First, we did not scrutinize the etiology of non-ST/MT group. Instead in the analysis, we made an assumption that *Rickettsia* is not a major etiology in the non-ST/MT group. It is possible that SFGR such as *R. japonica*, *R. felis*, and *R. honei* are causative agents in this group.^3^ In a preliminary screening of 132 available samples from the non-ST/MT group with an IFA with *R. japonica* antigen (strain YH), none of samples were clearly positive (S. Ando and others, unpublished data). This suggests that at least *R. japonica* is not a major problem. It is also not known whether this group consists of one dominant pathogen or a mixture of different etiologies such as other viral illnesses, leptospirosis, and Q fever.^13^,^39^,^40^ Prospective studies are necessary to reveal the true etiology of this group. Second, we used either a commercial IFA or an in-house IFA to diagnose murine typhus and then combined the results. In a small number of patients tested in both assays, the results were concordant. Third, in the current study, both scrub typhus and murine typhus were diagnosed only by serology. There is some controversy about the definition of serology diagnosis with IFA by a single blood sample for both rickettsiae.^19^,^41^ We chose the most frequently used cutoff value of 1:400 for diagnosis by single titer.

In conclusion, both scrub typhus and murine typhus are common in northern Vietnam. Several clinical symptoms and signs were associated with specific type of rickettsioses with high ORs. Such information should help local clinicians to make clinical diagnosis of specific rickettsioses. The combination of our results and the existing knowledge will reinforce clinical diagnosis of rickettsioses at a referral hospital in a resource-limited and malaria non-endemic area.

## Supplementary Material

Supplemental Table.

## Figures and Tables

**Table 1:** Demographic and clinical information of 579 patients with suspected rickettsial infection classified into those with positive serology results for scrub typhus or murine typhus

	Total *N* = 579 *n* (%)	Scrub typhus *N* = 237 *n* (%)	Murine typhus *N* = 193 *n* (%)	Non-ST/MT *N* = 149 *n* (%)
Basic information				
Age (mean, SD), years	46.2, 15.7	46.6, 16.9	47.1, 14.0	44.6, 15.7
Male gender	358 (61.8)	119 (50.2)	139 (72.0)	100 (67.1)
Living in Hanoi	241 (41.6)	67 (28.3)	102 (52.9)	72 (48.3)
Rainy season (May–October)	344 (59.4)	177 (74.7)	94 (48.7)	73 (49.0)
High-exposure occupation* (*N* = 575)	271 (47.1)	145 (61.2)	75 (39.3)	51 (34.7)
β-Lactam antibiotics ineffective (*N* = 244)	189 (77.5)	85 (70.8)	58 (82.8)	46 (83.6)
Symptoms				
Fever duration > 7 days	383 (66.2)	180 (76.0)	125 (64.8)	78 (52.4)
Headache	403 (69.6)	167 (70.5)	139 (72.0)	97 (65.1)
Myalgia	425 (73.4)	161 (67.9)	153 (79.3)	111 (74.5)
Rigor/chill	448 (77.4)	195 (82.3)	140 (72.5)	113 (75.8)
Cough	192 (33.2)	104 (43.9)	49 (25.4)	39 (26.2)
Sputum	46 (7.9)	24 (10.1)	13 (6.7)	9 (6.0)
Dyspnea	38 (6.6)	33 (13.9)	2 (1.0)	3 (2.0)
Abdominal pain (*N* = 568)	17 (3.0)	10 (4.3)	1 (0.5)	6 (4.2)
Diarrhea	89 (15.4)	37 (15.6)	30 (15.5)	22 (14.8)
Physical signs				
Body temperature > 38°C (*N* = 578)	457 (79.1)	191 (80.6)	151 (78.2)	115 (77.7)
Heart rate > 90/min (*N* = 569)	184 (32.3)	90 (39.0)	48 (25.0)	46 (31.5)
Relative bradycardia† (*N* = 569)	244 (42.9)	92 (39.8)	94 (49.0)	58 (39.7)
Respiratory rate > 20/min (*N* = 397)	131 (33.0)	73 (40.1)	34 (27.0)	24 (27.0)
Hypotension‡ (*N* = 577)	14 (2.4)	12 (5.1)	1 (0.5)	1 (0.7)
Altered mental status	30 (5.2)	23 (9.7)	4 (2.1)	3 (2.0)
Rash	217 (37.5)	74 (31.2)	86 (44.6)	57 (38.3)
Lymphadenopathy	215 (37.1)	152 (64.1)	38 (19.7)	25 (16.8)
Eschar	161 (27.8)	149 (62.9)	4 (2.1)	8 (5.4)
Hepatomegaly and/or splenomegaly	253 (43.7)	118 (49.8)	77 (39.9)	58 (38.9)
Edema	93 (16.1)	66 (27.9)	17 (8.8)	10 (6.7)
Lung rales	139 (24.0)	83 (35.0)	33 (17.1)	23 (15.4)
Laboratory				
Hematocrit < 30% (*N* = 570)	44 (7.7)	31 (13.2)	6 (3.1)	7 (4.9)
WBC > 10,000/μL (*N* = 576)	178 (30.9)	96 (40.7)	51 (26.6)	31 (21.0)
Platelet < 100,000/μL (*N* = 564)	254 (45.0)	104 (45.0)	80 (42.6)	70 (48.3)
AST or ALT > 40 IU/L (*N* = 475)	451 (95.0)	194 (97.5)	157 (96.3)	100 (88.5)
Total bilirubin > 3 mg/dL (*N* = 388)	21 (5.4)	13 (7.7)	4 (3.1)	4 (4.5)
BUN > 20 mg/dL (*N* = 551)	144 (26.1)	82 (36.4)	39 (21.0)	23 (16.4)
Creatinine > 1.5 mg/dL (*N* = 404)	31 (7.7)	19 (11.5)	6 (4.5)	6 (5.7)
Outcome				
Died (*N* = 578)	1 (0.2)	1 (0.4)	0 (0.0)	0 (0.0)
Defervescence > 3 days (*N* = 563)	119 (21.1)	50 (21.8)	36 (18.9)	33 (23.1)

ALT = alanine aminotransferase; AST = aspartate aminotransferase; BUN = blood urea nitrogen; non-ST/MT = non–scrub and murine typhus; SD = standard deviation; WBC = white blood cell.

* High-exposure occupation: occupation with frequent contacts with natural environment, such as farming, dairy husbandry, and environmental construction engineering.

† Relative bradycardia: less than 10 per minute increase in heart or pulse rate when the body temperature increased 1°C.

‡ Hypotension: systolic blood pressure < 90 mmHg or diastolic blood pressure < 50 mmHg.

**Table 2:** Statistical significance of clinical findings for scrub typhus, murine typhus, and non–scrub and murine typhus

	Scrub typhus		Murine typhus		Non-ST/MT	
	OR	*P* value	OR	*P* value	OR	*P* value
Basic information						
Age (mean, SD), years	1.02	0.7	1.06	0.3	0.90	0.1
Male gender	0.43	< 0.001	1.96	< 0.001	1.36	0.1
Living in Hanoi	0.38	< 0.001	1.99	< 0.001	1.44	0.06
Rainy season (May–October)	3.09	< 0.001	0.52	< 0.001	0.56	0.003
High-exposure occupation*	2.65	< 0.001	0.62	0.008	0.50	0.001
β-Lactam antibiotics ineffective	0.47	0.02	1.77	0.1	1.64	0.2
Symptoms						
Fever duration > 7 days	2.16	< 0.001	0.91	0.6	0.45	< 0.001
Headache	1.07	0.7	1.19	0.4	0.76	0.2
Myalgia	0.63	0.01	1.60	0.02	1.08	0.7
Rigor/chill	1.63	0.02	0.67	0.05	0.89	0.6
Cough	2.26	< 0.001	0.58	0.005	0.64	0.04
Sputum	1.64	0.1	0.77	0.4	0.68	0.3
Dyspnea	10.90	< 0.001	0.10	0.002	0.23	0.02
Abdominal pain	2.07	0.1	0.12	0.04	1.63	0.3
Diarrhea	1.03	0.9	1.02	0.9	0.94	0.8
Physical signs						
Body temperature > 38°C	1.17	0.5	0.93	0.7	0.90	0.6
Heart rate > 90/min	1.66	0.005	0.59	0.008	0.95	0.8
Relative bradycardia†	0.81	0.2	1.45	0.04	0.84	0.4
Respiratory rate > 20/min	1.81	0.006	0.66	0.08	0.69	0.2
Hypotension‡	9.01	0.004	0.15	0.07	0.22	0.1
Altered mental status	5.14	< 0.001	0.29	0.02	0.31	0.06
Rash	0.63	0.01	1.56	0.01	1.05	0.8
Lymphadenopathy	7.92	< 0.001	0.29	< 0.001	0.25	< 0.001
Eschar	46.56	< 0.001	0.03	< 0.001	0.10	< 0.001
Hepatomegaly and/or splenomegaly	1.52	0.01	0.79	0.2	0.77	0.2
Edema	4.50	< 0.001	0.39	0.001	0.30	0.001
Lung rales	2.75	< 0.001	0.54	0.006	0.49	0.005
Laboratory						
Hematocrit < 30%	3.76	< 0.001	0.29	0.006	0.54	0.1
WBC > 10,000/μL	2.16	< 0.001	0.73	0.1	0.51	0.003
Platelet < 100,000/μL	1.00	1.0	0.86	0.4	1.19	0.4
AST or ALT > 40 IU/L	2.87	0.04	1.60	0.3	0.24	0.001
Total bilirubin > 3 mg/dL	2.20	0.09	0.45	0.2	0.78	0.7
BUN > 20 mg/dL	2.44	< 0.001	0.66	0.05	0.47	0.003
Creatinine > 1.5 mg/dL	2.46	0.02	0.46	0.1	0.66	0.4
Outcome						
Defervescence > 3 days	1.07	0.7	0.81	0.3	1.17	0.5

ALT = alanine aminotransferase; AST = aspartate aminotransferase; BUN = blood urea nitrogen; non-ST/MT = non–scrub and murine typhus; SD = standard deviation; WBC = white blood cell.

* High-exposure occupation: occupation with frequent contacts with natural environment, such as farming, dairy husbandry, and environmental construction engineering.

† Relative bradycardia: less than 10 per minute increase in heart or pulse rate when the body temperature increased 1°C.

‡ Hypotension: systolic blood pressure < 90 mmHg or diastolic blood pressure < 50 mmHg.
