# Supplementary material for: Clinical and Epidemiological Characteristics of Scrub Typhus and Murine Typhus among Hospitalized Patients with Acute Undifferentiated Fever in Northern Vietnam
Source: Am J Trop Med Hyg. 2015 May 6;92(5):972–8. doi: 10.4269/ajtmh.14-0806 (PMC4426587; doi:10.4269/ajtmh.14-0806)
Supplement: Supplementary file 1 [file SD6.pdf]

Supplemental Table 1

Comparison of basic information between 579 included and 52 excluded patients

	Patients		
	Included, $N = 579$	Excluded, $N = 52$	P value
Age (mean, SD)	46.2, 15.7	52.2, 17.6	0.009
Sex	358 (61.8)	36 (69.2)	0.3
Hanoi	241 (41.6)	23 (44.2)	0.7
Rainy season (May–October)	344 (59.4)	36 (69.2)	0.2
High-exposure occupation*	271 (47.1)	23 (45.1)	0.8
β-Lactam antibiotics not effective	189 (77.5)	15 (83.3)	0.8
Rash	217 (37.5)	25 (48.1)	0.1
Lymphadenopathy	215 (37.1)	13 (25.0)	0.08
Eschar	161 (27.8)	9 (17.3)	0.1
Hepatomegaly/splenomegaly	253 (43.7)	22 (42.3)	0.8

SD = standard deviation.

*High-exposure occupation: occupation with frequent contacts with natural environment, such as farming, dairy husbandry, and environmental construction engineering.
